# How the COVID-19 Pandemic Impacted Medical Education during the Last Year of Medical School: A Class Survey

**DOI:** 10.3390/life11040294

**Published:** 2021-03-30

**Authors:** Gillian Franklin, Clare Martin, Marc Ruszaj, Maliyat Matin, Akaash Kataria, Jinwei Hu, Arlen Brickman, Peter L. Elkin

**Affiliations:** 1 Department of Biomedical Informatics, Jacobs School of Medicine and Biomedical Sciences, University at Buffalo, Buffalo, NY 14203, USA; arlenbri@buffalo.edu (A.B.); elkinp@buffalo.edu (P.L.E.); 2 Jacobs School of Medicine and Biomedical Sciences, University at Buffalo, Buffalo, NY 14203, USA; claremar@buffalo.edu (C.M.); marcrusz@buffalo.edu (M.R.); maliyatm@buffalo.edu (M.M.); Akaashka@buffalo.edu (A.K.); Jinweihu@buffalo.edu (J.H.); 3 Department of Veterans Affairs, VA Western New York Healthcare System, Buffalo, NY 14215, USA; 4 Faculty of Engineering, University of Southern Denmark, DK-5230 Odense, Denmark

**Keywords:** novel coronavirus disease 2019, COVID-19, medical education, telemedicine, telehealth, E-learning, tele-education

## Abstract

The novel coronavirus disease 2019 (COVID-19) pandemic has changed the medical education platform for students in the United States of America (USA). In that light, medical schools had to rapidly rearrange the dynamics of their educational curricula from the traditional platforms, to incorporate telemedicine. The telemedicine platform is supported in many specialties, allowing students various options to continue their education without interruption during the COVID-19 pandemic, and beyond. Telemedicine platforms are projected to grow exponentially due to the COVID-19 pandemic, allowing a segue for medical schools to modify their curricula by incorporating telemedicine programs. These distant-, e-learning (tele-education) programs align with the recommendations and guidelines for practicing social distancing. In this article, we surveyed fourth-year medical students to better understand their views on multiple aspects of e-learning, and its impact on their medical education during the COVID-19 pandemic. We assessed the medical students’ experiences, satisfaction, insight and knowledge with e-learning, tele-education, telehealth, and their related modalities during COVID-19. We provide an organized overview and analysis of the main factors that influence medical education during the COVID-19 pandemic, while bringing forth the main challenges, limitations, and emerging approaches in the field of telemedicine and its application as it relates to medical education and e-learning across medical specialties. We outline the main themes and ideas that the medical students voiced, as to how their medical education is being impacted by the COVID-19 pandemic and how they will incorporate telemedicine and tele-education in their future career. A cross-sectional, mixed-method survey was developed and distributed via Google Surveys to 181 University at Buffalo, Jacobs School of Medicine and Biomedical Sciences, United States of America, 4th year medical students, in December 2020. Results were compiled and analyzed after a 6-day open period for responses to be submitted. The survey instrument consisted of questions that inquire about the students’ perspectives as it relates to their rapid switch from their traditional method of learning to the on-line version of medical education during the COVID-19 pandemic. A total of 65 students responded to the survey, of which 63 completed the survey. More than half of the students (*n* = 63, 57%) indicated that both their specialty of interest, and (*n* = 21, 33%) their sub-internships were impacted by the temporary lockdown, due to the COVID-19 pandemic. Students also indicated that the top three specialties that were affected included surgery, internal medicine and obstetrics and gynecology. When the students were asked if they were satisfied with the use of aquifer for their health care e-learning, only 35% of the students were satisfied. The students expressed that the school’s administration team did a good job in developing the new tele-education curriculum for those in clinical training. In addition, responses indicated that students were open to case-based video learning and readings, when combined with the abbreviated clinical exposure during the make-up “clinical immersions periods” allowed for adequate learning. Overall, the survey responses show that more than half, approximately 54% of the medical students utilized telemedicine platforms during their clerkships that were impacted by COVID-19. The 4th-year medical students did not find tele-education and e-learning to be as effective as traditional medical education that combines in-person didactic classroom instructions and in-person face-to-face in hospital clerkships. Students felt that the telemedicine program that was rapidly set up due to the COVID-19 ‘lockdown’ was fragmented, since it was not a formal integration of a telemedicine E-learning program. Students would have preferred more ‘real’ cases to follow, instead of the ready-made, aquifer type of cases. Telemedicine has significant potential to address many of the challenges facing the medical education environment today. We believe now that people have become comfortable with this method of teaching, that even after the pandemic ends, we will continue to see tele-education used as a platform for medical education.

## 1. Introduction

In the advent of a novel coronavirus, the severe acute respiratory syndrome coronavirus 2 (SARS-CoV-2), responsible for the coronavirus disease 2019 (COVID-19) [1,2], the World Health Organization has declared a global pandemic [3]. During these unprecedented times, the terms telemedicine and telehealth have become popularized, although they have been known and used for decades [4,5,6]. The use of these technologic modalities has risen to an insurmountable level as tools for limiting in-person interactions between health care providers and patients. Telemedicine has become “not just a novelty, but a necessity” [7]. The World Health Organization has rendered telemedicine an essential service considering the COVID-19 pandemic [8]. Although the terms telemedicine and telehealth are synonymous, and are used interchangeably, their definitions and scope vary. According to the American Telemedicine Association (ATA), “telemedicine is using electronic communications to exchange medical information from one area to another in order to improve patients’ health status”, and telehealth effectively connects individuals and their healthcare providers when an in-person interaction is not clinically necessary and facilitates physician-to-physician consultation” [7,9]. Bashshur defines telemedicine as an integrated healthcare delivery system in which computer technology and telecommunications are utilized in place of face-to-face communication between patient and provider [10], while the United States Department of Health and Human Services (HHS) defines telehealth as the use of electronic information and telecommunications technologies, such as a telephone or the internet to deliver health-related services when the patient and doctor are not in the same location at the same time [11]. Overall, telehealth is an umbrella term that incorporates the use of digital or electronic information and communications technologies to compliment patient and professional health education, clinical health care, health administration and public health, generally improving healthcare as a whole [6,12], while telemedicine is more focused on communications via electronic devices for services such as follow-ups, specialist consultation and medication management [6].

Telemedicine became more commonplace, not only in clinical settings, but also in medical schools, as a modality for medical education during the COVID-19 pandemic. In this way, telemedicine is effectively offering medical schools a timely opportunity to incorporate this virtual method for training into the curricula of training future doctors [13]. Traditional medical education and training in the United States of America (USA) medical schools has abruptly changed due to the COVID-19 pandemic. On 17 March 2020, the Association of American Medical Colleges (AAMC) and Liaison Committee on Medical Education (LCME) proposed that medical students’ clinical rotations be postponed, to limit their direct patient contact in response to the COVID-19 pandemic [14,15]. Medical schools had to rapidly modify their curricula to incorporate alternative teaching methods for in-person didactic lessons in the preclinical years, as well as in face-to-face in healthcare settings clinical clerkships, where direct contact with patients was no longer allowed [16,17]. With social distancing standards and guidelines in place, technologies such as telemedicine/telehealth as a platform for interim medical education became one alternative to the traditional forms of medical education for training future physicians [13,14,15,16,17,18]. Medical students who were entering their fourth and final year of medical school were mostly impacted by these new regulations and they were thrusted into the world of remote learning. Remote medical education (RME), “e-learning” has heightened as a platform for medical education, and has now become part of the ‘norm’ in educating medical students [19,20,21]. With this abrupt change, medical schools moved to telemedicine platforms, which had to be rapidly instituted for the continuation of medical education. Throughout its history, the use of technology as platforms in medical school curricula has been limited [22,23]. However, telemedicine was integrated in medical education during the medical student’s clinical clerkships [13], a significant time in moving from classroom to face-to-face encounters with patients. According to Guze [24] (2015), the purpose of incorporating key telemedicine platforms in medical education is to “facilitate basic knowledge acquisition, improving decision making, enhancement of perceptual variation, improving skill coordination practicing for rare or critical events, learning team training, and improving psychomotor skills.” Jumerornvong et al. (2020), agrees that if these skills are incorporated into medical student curricula, it would prepare them for the pandemic on hand, as well as future ones. Telemedicine platforms are projected to grow exponentially due to the COVID-19 pandemic [25], allowing a segue for medical schools to modify their curricula by incorporating telemedicine programs for e-learning and tele-education. These distant-, e-learning and tele-education programs align with the recommendations and guidelines for practicing social distancing and ‘flattening the curve’ [15]. In this article, we surveyed fourth-year medical students to better understand their views on multiple aspects of e-learning and tele-education, and its impact on their medical education during the COVID-19 pandemic.

The goal of this study was to get a better understanding of how the COVID-19 pandemic impacted the fourth-year medical student learning, after a complete changeover from in-person to remote learning. We therefore assessed the medical students’ experiences, satisfaction, insight and knowledge with e-learning, tele-education, telehealth, and their related modalities during the COVID-19 pandemic. We provide an organized overview and analysis of the main factors that influence medical education during the COVID-19 pandemic, while bringing forth the main challenges, limitations, and emerging approaches in the field of telemedicine and its application as it relates to medical education and e-learning across medical specialties. We outline the main themes and ideas that the medical students voiced, as to how their medical education is being impacted by the COVID-19 pandemic and how they will incorporate telemedicine and tele-education in their future career.

## 2. Methods

We designed a structured cross-sectional, mixed-method survey in English, that contained fourteen questions related to medical education during the COVID-19 pandemic (see Appendix A). The University at Buffalo (UB) Institutional Review Board (IRB) approved this study’s protocol. An email invitation that included a pre-survey informed consent to complete an electronic version of the cross-sectional analysis mixed-method survey (https://docs.google.com/forms/d/e/1FAIpQLSc-UPeKfLy3p2K68OkZEllv2kfHX3mu4LhoK0sec_0rKxu5ow/formResponse) (accessed on 22 December 2020) was distributed via Google Surveys, in December 2020. The survey was sent to eligible participants, 181 (84 females, 97 males, as per UB registrar) fourth year medical students at the UB, Jacobs School of Medicine and Biomedical Sciences, Buffalo, New York. This was a convenience sample size of all fourth-year medical students, allowing for the expeditious data acquisition.

For face validity, the survey instrument was reviewed by fellows and attending physicians in the clinical informatics department at the University at Buffalo, prior to distribution to the 4th year medical students, to ensure that the questions were clear, well-structured and well-rounded [26]. The survey questions were in a mixed-method style, including Likert-like scale and, dichotomous yes/no questions, as well as open layout questions for written responses. Some survey questions specifically referred to ‘Aquifer,’ this is a non-profit organization that delivers health care education to students, using simulated patients, through a guided case modules interface [27].

The questions for the survey instrument were sent to a current 4th year medical student who uploaded the questions into Google Forms, the online platform that was chosen to deliver the self-administered surveys. The surveys were distributed via the link above. An informed consent statement preceded the survey instrument. Participation in the survey responses was voluntary, free and anonymous. There were no incentives such as gifts in monetary form or otherwise. All consenting participants were allowed to proceed to complete the self-administered survey in December 2020. After a 6-day open period for responses to be submitted, the survey was disabled and responses were downloaded into a Microsoft Excel spread sheet for review and analysis. Analysis was also done in the Google software platform. The survey questions are in Appendix A.

Responses to the survey questions were analyzed and compiled into graphs by google survey software. Responses related to the impact of COVID-19 on medical education was compiled and analyzed using methods of grounded theory and qualitative analysis as described in Babchuk’s “Fundamentals of qualitative analysis in family medicine [28].” Responses to the open-ended question “Please share any thoughts you have regarding how your education was impacted and what you see as a feasible solution if a second wave of COVID should arise,” and other open-ended questions were reviewed multiple times individually by two independent researchers (CM and GF). Responses were coded line by line with key summarizing phrases, individually. Codes were then grouped into themes and subthemes, which were compared among researchers and an established set of themes were agreed upon. These themes were then further organized into categories and grouped with supporting quotes from the responses which supported the themes. Lastly, themes/subthemes and quotes were consolidated and organized into paragraph form for presentation [28].

## 3. Results

### 3.1. Quantitative Analysis

This cross-sectional study was conducted among 181 medical students, 84 females and 97 males, in their 4th year of medical school, enrolled in the University at Buffalo, Jacobs School of Medicine and Biomedical Sciences. Of the 181 students, 65 (36%) responded to the survey, of which 2 (3%) opted out, and 63 completed the survey. Table 1 depicts the students’ breakdown by clerkship specialty that were impacted by the COVID-19 pandemic. Fifty-seven percent (36/63) indicated that their specialty of interest was impacted and 21/63 (33%) indicated that their sub-internship was affected.

In Table 2, the first column illustrates the number of students involved in the use of telemedicine for patient care by specialty and in their sub-internship. The top 3 specialties in which telemedicine modalities were most used include family medicine (32%), internal medicine (29.4%), and pediatrics (15.8%). The last column of Table 2 shows the number of students who use aquifer, a guided simulated case modules interface used for clinical learning, as their primary clinical experience. Surgery (96%), internal medicine (91.4%), and obstetrics and gynecology (87.5%) were the top 3 clerkship specialties in which the students used this modality as their main source for clinical learning. Of those students who answered “yes,” a subsequent question was asked about their level of satisfaction with this particular modality, on a Likert-like scale from 1–5, with 1 being highly unsatisfied and 5 being highly satisfied. Twenty-one of sixty students (35%) were satisfied with the use of aquifer as a source of clinical learning, while 2/60 (3.3%) were highly satisfied, 16/60 (26.7%) were mostly satisfied, 16/60 (26.7%) were mostly unsatisfied, and 5/60 (8.3%) were highly unsatisfied.

**Figure 1:**
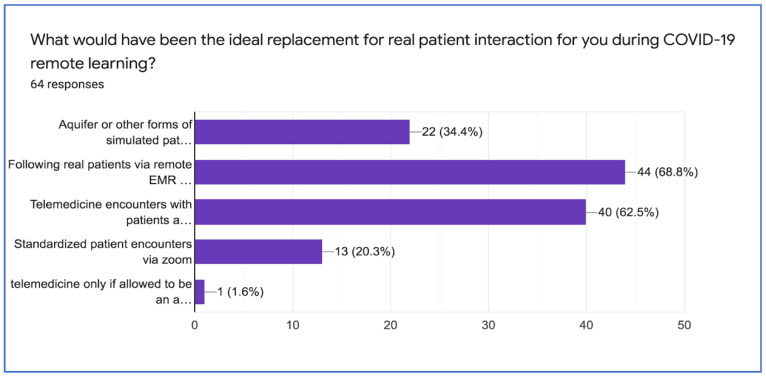
Ideal replacement for real patient interaction during COVID-19 pandemic.

The students were asked about their “ideal” replacement for ‘real’ patient interaction during their tele-education during the pandemic. Figure 1 depicts their responses. Most students 44 (69.8%) would have preferred “following real patients via remote electronic medical record (EMR) and presentations at virtual rounds with and inpatient team, while of 40 (62.5%) of students would have preferred telemedicine encounters with patients and preceptor, followed by 22 (34.4%), who would have preferred aquifer or some other form of simulated patient experience.

Table 3 shows the results of a question asking students if the education that they received in their clerkships that were impacted by the COVID-19 prepared them for the following; most of the student respondents felt that the medical education that they received through tele-education and e-learning prepared them well for their shelf examinations (*n* = 63, 76%), and the United States Medical Licensing Examination (USMLE) Step 2 clinical knowledge (CK) examination (*n* = 63, 76%). However, only 23/63 (37%) felt prepared for their internship year.

The medical students were asked if they believed that the university is prepared to offer an adequate educational experience, if there is a second wave of COVID-19, and more than half of the students 42/63 (66.7%) answered “yes,” while 21/53 (33.3%) answered “no.” We asked students about telemedicine platforms such as using iPads, and their potential usefulness as a resource to connect to patients and their team virtually, if they were available in an inpatient or outpatient setting during COVID-19. Again, for the inpatient setting, more than half 41/62 (66%) of the students responded “yes,” while 21/62 (34%) responded “no.” Additionally, for the outpatient setting, 42/62 (68%) of the students responded “yes,” and 19/62 (31%) answered “no.” The students were asked if telemedicine were to be utilized in response to the COVID-19 distance learning, would they like a brief training course to learn how to facilitate telemedicine visits. There was an overwhelming response 58/63 (90.6%) answered ”yes.” Overall, the survey responses show that approximately 54% (34/63) of the medical students who utilized telemedicine during their clerkships were impacted by COVID-19.

### 3.2. Qualitative Responses (Excerpts)

Students responded that they used both telephone and video-conferencing platforms to observe and actively participate in telemedicine in the outpatient setting, in addition to “virtual rounding” via remote access to the Electronic Medical Record (EMR) in the inpatient setting. About one third of the students who used telemedicine 11/34 (32.4%) reported that they only observed their attending or resident interviewing patients via telemedicine and did not directly interact with patients. A group of students 8/34 (23.5%) reported they performed patient interviews which were observed by resident or attending and then they presented to their attending. *“Would call a patient then present to the doctor and she would call them back and we’d talk together. Or sitting watching residents do telemedicine calls.”*

A smaller number of students 4/34 (11.8%) stated that their telemedicine experience included “virtual rounding” on patients that they followed via remote access to the Electronic Medical Record (EMR). Using this method, they would gather information from the chart including history, physical exam, vitals, labs, and come up with a plan to present to their attending via video conference. Often these various modalities were used in combination: *“My sub-I was virtual over zoom. Students watched attendings call their patients over the phone and listened in on their conversations. Students were also given remote EMR access to follow along on the computer.”*

We went on to ask students to share any thoughts regarding how their education has been impacted and what they foresee as a feasible solution in the event that there is a second wave of COVID-19; the students’ thoughts were grouped in themes/subthemes in Table A1. Words below (see Appendix A)

*“Jacobs School of Medicine and Biomedical Sciences (JSMBS) did a good job considering the circumstances.”*

Overall, medical students expressed that the school administration did a good job comprising a new curriculum for the clinical hiatus during the first wave of the COVID-19 pandemic, considering the difficult circumstances and limited time available. Students were pleased with the diversity of experiences made available to them including Aquifer (patient simulation, using a guided case modules interface for clinical learning), videos for content review, and small group sessions with their peers and attendings. They appreciated the intense pressure administrators were under to produce a meaningful replacement in such a short amount of time. A few students commented that clerkship leaders were helpful and responsive to feedback which included reducing the amount of Aquifer case learning content. *“Surgery clerkship leader (Dr. M) did a good job in response to generate a curriculum. WISE-MD was very helpful. She also got feedback from us to try to reduce the amount of Aquifer and cut out a few of the low-yield faculty lectures.”*

Responses indicated that students thought that the case-based video learning and readings, when combined with the abbreviated clinical exposures during the make-up “clinical immersion periods” allowed for adequate learning. *“Although nothing replaces real in-person interactions, I believe that the Aquifer cases and assigned videos/readings were adequate to learn the necessary material and then the shortened clinical immersion experiences gave the opportunity to apply that learned material.”*

A few students shared that the amount of communication from the Office of Medical Education was lacking during this time period. Although it was acknowledged that circumstances were constantly changing during this time, students felt they could have benefited from more frequent and clear communication from the deans. Other students commented that there should have been a more consolidated approach for all remote learning as there was apparent variation and discrepancies in types of experiences available and amount of work on different clerkships. *“There should be a unified approach throughout all clerkships. Try to get students to participate in rounds virtually and do Aquifer cases as well. I think those 2 options are the best.”*

When asked about the ‘desire for replacement, something that’s better than Aquifer cases,’ students had a common theme that if a second wave of COVID-19 were to occur, students would desire a more robust experience than relying on Aquifer case learning modules. Students felt that assigning these videos was just “adding another chore to perform” and they did not think that videos were engaging in a way that simulates real patient interactions. “I would really like to see the university not rely as much on aquifer for our education should there be a second wave. The cases were too easy to click through and disengage from, and third year is normally so clinical and so engaging it felt like I wasn’t even in medical school.” “I think clerkship administration did the best they could in response to COVID. However, since they could prepare more for a second wave, methods of learning other than Aquifer would be useful, such as telemedicine visits, following patients virtually through EMR, etc.” Following patients virtually via remote Electronic Medical Record is a feasible option.

One form of remote learning that was reportedly utilized on the pediatric clerkship and sub-internships was the ability to have remote access to the Electronic Medical Record (EMR) to follow patients, perform chart reviews, obtain history, vitals, labs, etc. and synthesize this information into a presentation that was given to attending physicians. This method of e-learning received excellent feedback from medical students, who felt it provided the best real-life experience they could have hoped for during tele-education. *“I felt following patients* via *EMR was most effective. This was essentially my daily exercise prior to COVID except it leaves out the physical exam, which can’t be replaced regardless. It’s as close as you can get to the real thing.”*

Following patients virtually allowed for exposure to their complex histories, atypical presentations, long medication lists, and other such innate components of learning medicine that are notably missing from the online cases which may oversimplify such elements. After researching their patients and presenting the cases to the attendings, students were able to engage in organic discussions with their team which provided enhanced opportunities for learning. *“I thought that following patients virtually was a great experience because we were given time to research the patients and their conditions making us better prepared for the team conversations about them and giving us the opportunity to learn more.”*

Some students did acknowledge that although this method of learning had good potential, it was not executed very well, because students could take shortcuts that prevented them from learning by taking information from the progress notes. *“On pediatrics, when we would chart check patients and then present them in the afternoon to attendings, I feel like the idea was right, with having us read about real patients, but the actual presenting wasn’t helpful because we could essentially just read off yesterday’s progress note written by a resident, so we weren’t doing any thinking.”*

### 3.3. In Person Learning Is Best

Medical students were clear that to a great extent, there is no good replacement for in-person learning during their third-year clinical clerkships. Exposure to, and direct involvement in patient care in real time encompasses a crucial component of their learning and preparation for residency. Some students felt that being so close to graduation, medical students should be kept in rotations for as long as possible despite infectious concerns because in a few short months they will be expected to function as competent residents, pandemic or not. One student commented that once they had the opportunity to be working in hospitals during the second wave of COVID, they benefited greatly from the exposure they received by witnessing how healthcare fundamentally changed during a pandemic. Such experiences brought rich context and unique perspectives to their medical education, and allowed their participation in perhaps the most historic time in their medical careers.

“*I think that neuro rotation also gave me better exposure to learning how patient care works during a pandemic. Same with my surgery sub-i. It was there that I saw the frustration of forgetting to order the COVID test, faced the struggle of wearing N95 for 8 h during an emergency case, and even saw some vascular complications of COVID, and also was able to see teams work covid into their ddx’s where it wouldn’t have been previously. I saw none of this while I was reading aquifer cases.*”

“*The in-hospital learning was the only thing that was affected that mattered. Book learning which mainly occurs through Uworld and anki wasn’t. A feasible solution would be to allow medical students to still do rotations even though there will be less volume, it is better than anything else. If students find it too risky and wish to sacrifice their education, that is on them.*”

### 3.4. Shortcomings of Remote Learning

Although remote learning may have been sufficient for students to learn the necessary material to pass their exams, there were a few common areas identified where lack of in-person attendance to rotations fell short of providing adequate medical education. First, multiple students stated that learning and developing practical and procedural skills suffered. This includes surgical skills in the operating room and various outpatient procedures/physical exams. It was suggested that if COVID-19 precautions should force schools to send students home again, that simulation centers be utilized in social-distanced manner so that students could continue learning and practicing such technical skills. *“I do feel like I missed out on some practical skills because of COVID. I think more in person simulations, if possible if the school does not close, would be helpful if we can’t be in hospitals.”*

“*I think my outpatient gyn procedure skills suffered as a result of remote learning because I couldn’t see actual patients and perform procedures. I only did a model once with the clerkship director but I didn’t feel like that was enough for me.*”

In addition, it was expressed that students were impacted by lack of resident/attending teaching during bedside rounds. Although virtual cases may provide necessary information for learning the salient take-home points for different diagnoses, students could not benefit from the stimulating conversations and learning that stemmed from such enlightening discussions during rounds. *“I really felt most engaged during the family medicine morning rounds with Dr. C. She would ask us questions about the disease process at random, and as much as it was stressful, you couldn’t disengage.”*

### 3.5. Fears

Along with shortcomings, medical students expressed concerns and fears regarding the impact of their interrupted education during the COVID-19 pandemic, including a delayed graduation if another wave of COVID should force them out of clinical rotations again. Many students did not feel the experiences during impacted clerkships adequately prepared them for intern year, and this was something they feared and reflected on during residency interviews with potential future employers. *“I have been asked a few times on interviews how my education was affected by covid, and I made the mistake of telling the first place I only have 8 days of actual in person peds clinical experience and 8 days of family med in person clinical experience and I think I just scared the crap out of them hiring me.”*

Other concerns expressed by students were about the fears of practicing and learning medicine during a pandemic if the situation should worsen and students are expected to fill in the gaps of care and are not in positions to advocate for their safety and wellbeing. Although no student reported running into any problems with this, a few students mentioned that they were grateful when attendings and residents prioritized their safety and made sure they had the necessary personal protective equipment (PPE), namely N95 masks, as it was sometimes tough to convince other hospital employees that medical students needed them. “*When my N95 was crushed and pretty much ruined, it was really key that one of the attendings was assertive in securing a new one for me from the NICU, because I had had issues when I went down by myself to get a new one. Understandably so—I know PPE is in high demand. But it can be tough to advocate for yourself when you’re a med student on the wards and some folks don’t think you need one.”*

*“Biggest fear is using medical students as free labor to deal with staffing concerns. If we are kept in the clinical environment it must be because it is truly safe and not because the hospitals need more bodies. School leadership needs to advocate for our wellbeing and work on building parallel curricula in the event that we are pulled out of in person learning. “Telemedicine is reasonable and has potential to be valuable part of education.”*

Numerous responses indicated that medical students are in favor of telemedicine as a viable solution to medical education during COVID-19, and a highly valuable skill that can be learned and applied in their future medical careers. Students are excited about telemedicine being incorporated into the standard of care for their specialty fields, and look forward to an opportunity to see how such technology can be utilized in a patient friendly manner. “*I think telemedicine would be amazing to introduce into our education. Especially since post-COVID most are predicting this will become standard practice... I also think we could have done more educating students on COVID and what healthcare looks like in the midst of a pandemic.”*

Students reported that they would greatly appreciate a brief educational course and specific training in ‘practicing’ telemedicine, so that they can become a part of in this exciting way of expanding access to care and reducing risk for their patients. They specifically expressed wanting to learn about different methods and technologies of performing a physical exam with telemedicine and telehealth technologies.

“*I think learning how telehealth works and even how to do as close as you can to a physical exam over telehealth would be INCREDIBLY valuable. I wish I could have tuned in to some pediatric telehealth visits and just seen how they did the exams!* Valuable to have the “*clinical experience” but also telehealth will likely become more and more important in our clinical arsenal, as I don’t think this will be the last pandemic we face in our careers.*”

It was proposed that telemedicine could serve as a feasible solution to many of the challenges posed by learning medicine during a pandemic. For example, following patients via remote EMR combined with virtual rounding with resident and attending teams would provide a stronger educational experience, and allow students to experience many of the elements that were highlighted as missing during the first wave of e-learning. *“I think telemedicine is a reasonable option but both faculty and students need to be taught how to make this happen. Students should not just be silent observers on telemedicine calls.”*

“*The only reasonable response to a second wave of COVID-19 would be to implement a telemedicine care model* via *iPads to facilitate patient interaction whilst maintaining appropriate distance.*”

## 4. Discussion

The goal of our study is to look at the impact of the COVID-19 pandemic on fourth-year medical students’ education. In recent times, there has been a big shift in the way medical education is conducted in the USA. There was a sudden and complete transition from in-person, “face-to-face” to remote learning modalities. This was necessary to maintain physical distancing, to limit the spread of COVID-19 caused by the severe acute respiratory syndrome corona virus 2 (SARS-CoV-2) [29,30]. Most medical schools halted their traditional format of teaching, with in-person didactic instructor-led lessons [31] and in-healthcare settings clerkship with “face-to-face patient” contact, to e-learning and tele-education, that incorporates telemedicine and telehealth technologies. Although some authors suggest that this style of medical education generally allows a more dynamic, interactive way of learning [31], the fourth-year medical students surveyed in this study, had mixed and varied feelings about that. Using telemedicine as a platform for medical education allow students to be a part of join inpatient rounds and even telemedicine visits and allows for presentations to faculty, as well as follow-up during the pandemic. However, with the rapid changeover from the traditional platform of learning to e-learning, students felt that some educational opportunities were lacking with the remote learning platform. Again, although televisits are in line with the social distancing guidelines, are free of COVID-19 risk, and provide outstanding safety for our medical students, our results indicate that the online learning platform could have been done somewhat differently. Telemedicine/telehealth will continue to slowly transform the way that medical education, including clinical clerkships training are delivered. These tools have the potential to improve patient access to specialty and subspecialty expertise, reduce healthcare costs, and improve the overall quality of medical education, as well as the medical student experience for a smooth transition to the workforce [26]. In a personal zoom interview with one of the 4th-year medical students [32], we learned that students had to intentionally enroll in additional electives rotations in order to obtain a satisfactory level of self-confidence needed to begin their internship year. This is a sensible strategy given the fact that the students lost some meaningful learning opportunities from in-person patient interactions, and this is where e-learning via telemedicine and telehealth come in handy. Although there are limitations to educating medical students using a virtual format [33], some of the physical examination for example, are not possible over a televisit. However, there have not been many articles in the literature discussing the negative effect to on patient care from this limitation. Telemedicine has tremendous potential to address many of the challenges facing the medical education environment today. It is well known, that in recent years, many professors began “flipping” the classroom to provide on-demand learning [30]. It is our belief that with the recent advances in using telemedicine and telehealth as platforms for e-learning in medical education, people will become more comfortable with this method of teaching, and the effect of moving from a traditional format of teaching to tele-education, and e-learning will continue long after the COVID-19 pandemic ends. We will continue to see tele-education as a part of medical education. E-learning and tele-education are essential during these times of COVID-19 pandemic, and their impact is significant for future life-long learning [31].

### 4.1. Strengths

The motivation for the survey was to gather and obtained information and insight to the successes and shortcomings that surfaced after the rapid switch from in-person, face-to-face to a remote learning experience during the early phases of the COVID-19 pandemic. We hope that the information gathered and compiled from the fourth-year medical student survey at the University at Buffalo, can subsequently inform and offer administrators and medical educators real-time, evidence-based information that can be instrumental in guiding best practices for remote curriculum redesign and development, during the lasting phases of the COVID-19 pandemic and beyond. As mentioned by other authors conducting similar research, this was not a hypothesis-driven study [34], the work from this survey is to be used as a guiding framework, and for streamlining more effective ways of teaching medical students and incorporating decisions and directions in their medical education e-learning curricula. The COVID-19 pandemic offers new opportunities in education for medical students [35].

### 4.2. Limitations and Future Research

Our cross-sectional survey study was limited by the small sample size, with only a 35% (63/181) response rate from the 4th-year medical students, a logistic constraint due to the existing number of enrolled (181) of fourth-year students [34]. However, given that this was a mixed method survey study, with qualitative open-text responses, a known limitation for survey-type studies [36], a lower response rate is acceptable [37]. Additionally, the response rate is not different from other survey studies conducted in medical schools during the COVID-19 pandemic, that reported a response rate among their student population as 53.7% for first-year students and 23.9% for second-year students [34]. With these shortcomings, the results of this study may not be generalizable to the entire class. Nonetheless, the student responses give us a general insight to how medical students view e-learning. The results of this study may be helpful with guiding future redesign for e-learning curricula in medical schools. According to Saleh and Bista [38], survey response rates correlate with participants’ survey completion rewards. In the future, with on-line survey studies like this one, offering an incentive such as bonus points on class assessment (quizzes and tests), or vouchers for free refreshments (coffee, tea, bottled water, sodas, etc.), food (pizza or lunch), school paraphernalia (hoodies, T-shirts, hats, mugs, keychain, etc.) [39], may be a good way to improve student participation. Similarly, there was limited time to change the entire fourth-year student curriculum towards an exclusive e-learning platform, therefore, the medical school defaulted to existing on-line and other videoconferencing platforms [34]. As mentioned in other studies [26,34], our survey results indicate that 4th year medical students’ feelings about e-learning varied, from some feeling that they are prepared, while others feel not so confident about moving on into their internship years. One of the major findings of this cross-sectional study, is that some of the medical students felt that they were more prepared for their United States Medical Licensing Examination (USMLE), that leads them to become licensed physicians. others felt less prepared with the switch to exclusive e-learning. However, the students did not feel that they were prepared to begin their internship year, because they missed a significant 3-month period during their 3rd year of medical school. These findings are similar to others in the literature [26,34]. Overall, the fourth-year medical students felt that the COVID-19 pandemic had a negative impact on their medical education, with significant limitations on “face-to-face” learning, specifically with patient interactions and their sub-internships, a limitation for beginning future training [26,34,35]. A systematic process for the survey instrument’s content validity was not addressed prior to its dissemination. We realized in retrospect that this was another shortcoming that would have helped us better as a checkpoint for relevance and effectiveness for the given target population [40]. However, the face validity of the survey instrument is not lacking, as it was reviewed by fellows and attending physicians in the clinical informatics department at the University at Buffalo, prior to distribution to the 4th year medical students. Additionally, our survey did not contain demographic questions. Therefore, we were unable to discuss the impact of age, race, ethnicity, socioeconomic status on biases or the survey responses. We were able to obtain the gender information from the UB registrar. Moreover, we did not conduct regression analysis to look at correlations and associations or the impact of e-learning on medical student remote curricula. We did not ask questions about the type of technical devices the medical students used (laptop computers, tablets, desktop computers, smartphones or iPads), Which may have given us some insight to the varied use of digital technology among medical students. Finally, in a pandemic situation, when policies are rapidly changing to protect communities from a spreading viral infection, there is probably not one way to deal with the issues at hand in medical education. As the COVID-19 pandemic slows down, medical schools will adapt to and incorporate new and more flexible forms of e-learning to their medical student curricula. This study should be replicated in future research with a survey that has been validated and piloted, as well as obtaining a larger sample size.

## 5. Conclusions

Overall, the rapid adaptation of online e-learning using various learning modalities such as Aquifer, from “face-to-face” in person, didactic learning, was useful to the fourth-year medical students at the Jacobs School of Medicine and Biomedical Sciences at UB. However, some students felt ill-prepared and less confident in moving into their next phase of graduate medical education, similar findings to other studies in the literature [26]. Incorporating, telemedicine and telehealth into medical student curriculum has the significant potential to address many of the challenges facing students and their medical education and training in these times of COVID-19 and beyond. Understanding medical students’ experiences and some of their unmet needs, during the COVID-19 pandemic (their last year of medical school), will enable administrators, teachers, and other staff to better support medical students moving forward, should there be a similar situation [41]. Based on the results of this cross-sectional survey study and consistent with what we observe daily, telemedicine, telehealth, e-learning and tele-education is going to be advanced by medical schools, especially those with medical students who are vocal and passionate about their medical education, as it relates to telemedicine. The challenges remain in validating its impact on medical education system and outcomes with scientific rigor, as well as in standardizing the methods used to assess cost-effectiveness. Telemedicine has significant potential to address many of the challenges facing the medical education environment today. We believe that with the recent advances in conducting medical education in a remote format, people have now become more comfortable with this method of teaching, a method that will extend beyond the end of the pandemic. For years to come, we will continue to see telemedicine and telehealth used as a platform in medical education. Therefore, now may be a good time to begin training students and faculty alike in using these modalities.

## Figures and Tables

**Table 1:** Clerkship impacted by COVID-19.

Specialty	Response *n* (%) N = 63
Surgery	6 (9.5)
Internal Medicine	13 (20.6)
Pediatrics	4 (6.3)
Obstetrics and Gynecology	1 (1.2)
Psychiatry	2 (3.2)
Family Medicine	2 (3.2)
Other (EM, Anesthesia, etc.)	8 (12.7)
Sub-internship	21 (33)

**Table 2:** Use of telemedicine vs. simulated case modality as primary patient care experience.

	Telemedicine Patient Care Involvement	Used Aquifer Simulated Cases as Primary Clinical Experience
Specialty	Response *n*/Total (%)	Response *n*/Total (%)
Surgery	1/25 (4.0%)	22/23 (95.7%)
Internal Medicine	10/34 (29.4%)	32/35 (91.4%)
Pediatrics	8/51 (15.7%)	24/27 (88.9%)
Obstetrics and Gynecology	1/26 (3.9%)	21/24 (87.5%)
Psychiatry	1/21(4.8%)	16/21 (76.2%)
Family Medicine	8/25 (32.0%)	18/24 (75.0%)
Sub-internship	5/25 (20.0%)	1/15 (6.67%)

**Table 3:** Preparedness with e-learning during COVID-19 Pandemic.

Questionnaire Item: Education Received during COVID Impacted Clerkships Adequately Prepared You for the Following? N = 63	Response *n* (%)	
	Yes	No
Self examinations	48 (76.2%)	15 (23.8%)
USMLE Step 2 CK examination	48 (76.2%)	15 (23.8%)
Internship year	23 (36.5%)	40 (63.5%)

## Data Availability

Not applicable.

## Appendix A

The following questions were included in the survey.

What clerkships were impacted by COVID for you? Were any of these your specialty of interest? If ‘Yes’, impacted by COVID, and/or ‘Yes’, to the ‘specialty of interest:’ The following choices of clinical specialties were listed to choose from (Surgery, Internal Medicine, Pediatrics, Obstetrics and Gynecology. Psychiatry, Family Medicine, Other specialty (Emergency Medicine, Anesthesia, etc. Sub-Internship?

Did any of your COVID impacted clerkships involve students in patient care via telemedicine? Yes/No

If yes, how was it done?

If YES, what was your level of satisfaction with telemedicine use in clerkships?

Likert scale 1-5 (highly unsatisfied to highly satisfied).

Did any of your COVID impacted clerkships use simulated cases (i.e., Aquifer) as the primary clinical experience? Yes/No

If YES, what was your level of satisfaction with this? Likert-like scale 1-5; 1 being ‘highly unsatisfied’ to 5 being ‘highly satisfied’.

What would have been the ideal replacement for real patient interaction for you during COVID-19 remote learning?

Aquifer or other forms of simulated patients?

Following real patients via remote EMR and presentation at virtual rounds with inpatient team

Telemedicine encounters with patients and preceptor

Standardized patient encounters via zoom

Other

If other? Your answer?

Do you feel the education you received during your COVID impacted clerkships adequately prepared you for the following? Yes/No to the following: Shelf exams, Step 2 CK, Intern year?

If iPads were available in the inpatient and outpatient settings during COVID-19, do you think this would be a useful resource to connect to patients and your team virtually? Yes/No: To the following: iPads in inpatient setting or iPad in outpatient setting?

How well do you feel the education you received during your COVID impacted clerkships taught you how to adapt patient care practices during a pandemic?

Likert-like scale 1-5, where 1 is ‘poorly prepared’ to 5 being ‘very well prepared’.

Do you believe that your university is prepared to offer an adequate educational experience if a second wave of COVID occurred? Yes/No

If telemedicine were to be utilized in response to COVID-19 distance learning, would you desire a brief training course to learn how to facilitate telemedicine visits? Yes/No.

Please share any thoughts you have regarding how your education was impacted and what you see as a feasible solution if a second wave of COVID should arise:

**Table A1:** Students’ thoughts on how their education is impacted and feasible solutions in the event that there is a second wave of COVID-19.

Themes/Subthemes	Student Quotes
In person learning is best Able to learn how medicine is adapted to pandemic times	*“I think students, especially those who will be graduating in less than 6 months should be kept in person as much as possible as they will soon be doctors, pandemic or not and must learn how to see patients so as not to compromise care as interns.”* “*I think that neuro rotation also gave me better exposure to learning how patient care works during a pandemic. Same with my surgery sub-i…It was there that I saw the frustration of forgetting to order the COVID test, faced the struggle of wearing N95 for 8 h during an emergency case, and even saw some vascular complications of COVID, and also was able to see teams work covid into their ddx’s where it wouldn’t have been previously. I saw none of this while I was reading aquifer cases.”*
Desire for better communication, more unified approach	*“We really need better communication with the deans. We never know what’s happening during this.”* *“There should be a unified approach throughout all clerkships. Try to get students to participate in rounds virtually and do aquifer cases as well. I think those 2 options are the best.”*
Areas missed out on: practical/procedural skills bedside rounds decreased time spent with patients	*“I do feel like I missed out on some practical skills because of COVID. I think more in person simulations, if possible if the school does not close, would be helpful if we can’t be in hospitals.”* *“Students are most impacted by the lack of resident/attending teaching because they miss bedside rounds, as well as increased time strain from COVID patients in general.* *I think my outpatient gyn procedure skills suffered as a result of remote learning because I couldn’t see actual patients and perform procedures. I only did a model once with the clerkship director but I didn’t feel like that was enough for me.”* *“I really felt most engaged during the family medicine morning rounds with Dr. Corliss. She would ask us questions about the disease process at random, and as much as it was stressful, you couldn’t disengage.”*
JSMBS did a good job with what they had; there is no good replacement	*“Surgery clerkship leader (Dr. M) did a good job in response to generate a curriculum. WISE-MD was very helpful. She also got feedback from us to try to reduce the amount of Aquifer and cut out a few of the low-yield faculty lectures.”* *“Overall, I feel the school did pretty well with the remote learning (aquifer and other guided cases, videos for content review, small group sessions) and with brief clinical exposure when it was safe to do so.”* *“I feel that UB did a great job implementing a virtual curriculum within a very timely fashion, within the context of a unique global pandemic. Although nothing replaces real in-person interactions, I believe that the Aquifer cases and assigned videos/readings were adequate to learn the necessary material and then the shortened clinical immersion experiences gave the opportunity to apply that learned material.”*
Desire for better replacement than Aquifer learning modules	*“I would really like to see the university not rely as much on aquifer for our education should there be a second wave. The cases were too easy to click through and disengage from, and third year is normally so clinical and so engaging it felt like I wasn’t even in medical school.”* *“Virtual stimulated cases like aquifer are poor replacements and become just another chore for students to perform.”* *“I think clerkship administration did the best they could in response to COVID. But since they could prepare more for a second wave, methods of learning other than Aquifer would be useful, such as telemedicine visits, following patients virtually through EMR, etc.”*
Fears: Not prepared for residency Using students as ‘free’ labor Not having appropriate PPE	“I have been asked a few times on interviews how my education was affected by covid, and I made the mistake of telling the first place I only have 8 days of actual in person peds clinical experience and 8 days of family med in person clinical experience and I think I just scared the crap out of them hiring me.” *“When my N95 was crushed and pretty much ruined, it was really key that one of the attendings was assertive in securing a new one for me from the NICU, because I had had issues when I went down by myself to get a new one. Understandably so—I know PPE is in high demand. But it can be tough to advocate for yourself when you’re a med student on the wards and some folks don’t think you need one.”* *“Biggest fear is using medical students as free labor to deal with staffing concerns. If we are kept in the clinical environment it must be because it is truly safe and not because the hospitals need more bodies. School leadership needs to advocate for our wellbeing and work on building parallel curricula in the event that we are pulled out of in person learning.”*
Telemedicine is reasonable and valuable part of our education Students should not be silent observers Education is a must	*“I think telemedicine would be an amazing to introduce into our education. Especially since post-COVID most are predicting this will become standard practice...I also think we could have done more educating students on COVID and what healthcare looks like in the midst of a pandemic.”* “*I think learning how telehealth works and even how to do as close as you can to a physical exam over telehealth would be INCREDIBLY valuable. I wish I could have tuned in to some pediatric telehealth visits and just seen how they did the exams! Valuable to have the “clinical experience” but also telehealth will likely become more and more important in our clinical arsenal, as I don’t think this will be the last pandemic we face in our careers.* *I think telemedicine is a reasonable option but both faculty and students need to be taught how to make this happen. Students should not just be silent observers on telemedicine calls.”*
Following patients virtually is a feasible option	“*I felt following patients* via *EMR was most effective. This was essentially my daily exercise prior to COVID except it leaves out the physical exam, which can’t be replaced regardless. It’s as close as you can get to the real thing.”* *Negative feedback:* “*On pediatrics, when we would chart check patients and then present them in the afternoon to attendings, I feel like the idea was right, with having us read about real patients, but the actual presenting wasn’t helpful because we could essentially just read off yesterday’s progress note written by a resident, so we weren’t doing any thinking*.” “*I thought that following patients virtually was a great experience because we were given time to research the patients and their conditions making us better prepared for the team conversations about them and giving us the opportunity to learn more.”*

“Jacobs School of Medicine and Biomedical Sciences (JSMBS) did a good job considering the circumstances”.
